# Divergent responses of the native grassland soil microbiome to heavy grazing between spring and fall

**DOI:** 10.1099/mic.0.001517

**Published:** 2024-11-26

**Authors:** Newton Z. Lupwayi, Xiying Hao, Monika A. Gorzelak

**Affiliations:** 1 Agriculture and Agri-Food Canada, Lethbridge Research and Development Centre, 5403 - 1st Avenue South, Lethbridge, Alberta, T1J 4B1, Canada

**Keywords:** cattle stocking rate, nutrient cycling, soil microbial diversity

## Abstract

Grasslands are estimated to cover about 40% of the earth’s land area and are primarily used for grazing. Despite their importance globally, there is a paucity of information on long-term grazing effects on the soil microbiome. We used a 68-year-old grazing experiment to determine differences in the soil permanganate-oxidizable C (POXC), microbial biomass C (MBC), the soil prokaryotic (bacterial and archaeal) community composition and enzyme activities between no-grazing, light grazing and heavy grazing, i.e. 0, 1.2 and 2.4 animal unit months (AUM) ha^−1^. The grazing effects were determined in spring and fall grazing. Light grazing had little effect on soil MBC and the composition and diversity of prokaryotic communities in either grazing season, but the effects of heavy grazing depended on the grazing season. In spring, heavy grazing increased the relative abundances of copiotrophic phyla *Actinomycetota, Bacillota* and *Nitrososphaerota*, along with soil POXC contents but decreased those of oligotrophic phyla *Acidobacteriota, Verrucomicrobiota* and *Nitrospirota*. This difference in responses was not observed in fall, when grazing reduced soil POXC, MBC and the relative abundances of most phyla. The *β*-diversity analysis showed that the prokaryotic community structure under heavy grazing was different from those in the control and light grazing treatments, and *α*-diversity indices (except the Shannon index) were highest under heavy grazing in both grazing seasons. The activities of P-mobilizing and S-mobilizing soil enzymes decreased with increasing cattle stocking rate in both seasons, but the activities of the enzymes that mediate C and N cycling decreased only in the fall. The genus *RB41* (phylum *Acidobacteriota*) was one of two core bacterial genera, and its relative abundance was positively correlated with the activity of the S-mobilizing enzyme. Therefore, light grazing is recommended to reduce negative effects on the grassland soil microbiome and its activity, and the grazing season should be considered when evaluating such grazing effects.

## Introduction

Grasslands are estimated to cover about 40% of the earth’s land area (excluding ice-covered areas) [1] and contain 20–30% of the global soil organic C [2]. In Alberta, grasslands cover 14.5% of the area [3] and are mainly used for cattle grazing. Grazing affects the soil either directly through trampling (which breaks up mulch layers and breaks soil aggregates, resulting in increased soil erosion) [4] and the addition of organic C and nutrients through dung and urine deposition [5], or indirectly by altering aboveground and belowground plant biomass [6]. These grazing effects depend on factors that include the stocking rate, forage type, climate and soil properties.

Among the soil properties affected by grazing is the soil microbiome, which may drive the functioning of the grassland ecosystem through biological processes such as nutrient cycling and biological pest control. Grazing has been reported to reduce soil microbial biomass [7,8] and increase the fungal–bacterial ratio because fungi tolerate periodic soil moisture fluctuations better than bacteria [8]. Eldridge *et al.* [9] reported that increasing grazing pressure reduced soil C, resulting in reduced relative abundance of *Actinomycetota*, the predominant bacterial phylum and increased relative abundance of *Ascomycota*, the predominant fungal phylum. Zhao *et al.* [10] observed that light and moderate grazing pressures did not affect the soil bacterial, fungal or total microbial abundances (determined by quantitative PCR), but heavy grazing intensity decreased them by 15–28 %. This distinction between intensities of grazing is important because many studies have shown that moderate grazing is better than no grazing in enhancing soil microbial processes and increasing grassland productivity by increasing belowground plant biomass relative to aboveground biomass [11,13]. Moderate grazing increased bacterial diversity, thereby maintaining soil stability [14]. Specifically, it increased the relative abundances of the bacterial classes *Nitrospirota*, *DA052*, *Syntrophobacteraceae*, *Methylocystaceae*, *Syntrophaceae*, *Mycobacteriaceae* and *Coriobacteriaceae *[12] reported that the genes that regulate soil C and N cycling increased under moderate grazing.

Grazing studies in the grasslands of Alberta have included effects on the composition of the vegetation [15], soil C storage [16], soil physical and chemical properties [17] and soil gas fluxes [18]. There is a paucity of information on grazing effects on the soil microbiome to compare with other grasslands around the world. Zhang *et al.* [19] reported that 64 years of very heavy grazing (4.8 AUM ha^−1^) reduced soil bacterial *α*-diversity and the relative abundances of *Bacteroidota, Chlorobi, Nitrospirota* and *Pseudomonadota* but increased the relative abundance of *Actinomycetota*. Their study did not evaluate soil functioning to link with the microbial composition. We used the same long-term trial to determine the seasonal effects (spring vs. fall) of light grazing (1.2 AUM ha^−1^) and heavy grazing (2.4 AUM ha^−1^) on the (a) composition and diversity of soil prokaryotic communities, (b) soil enzyme activities and (c) the core soil bacterial taxa in a native grassland of Alberta. We hypothesized that grazing intensity would affect these soil microbial properties, but the effects would be similar in the two grazing seasons.

## Methods

### Experimental site

The trial was conducted on a southwestern Alberta native grassland located in the Porcupine Hills of the Rocky Mountains (50°12′N, 113°54′W). It has rolling to hilly topography, with an altitude of 1420–1350 m above sea level. The soil is a Black Chernozem with clay loam to loam texture. The top soil layer (0–15cm) was tested in 2014 and found to have the following properties: pH (H_2_O): 6.18–6.51, organic C: 64.3–80.6 g kg^−1^ and total N: 6.3–6.9 g kg^−1^ [18]. The mean (1997–2013) annual precipitation and temperature were 494 mm and 5.3 °C, respectively, with more than 70% of the precipitation occurring during the growing season from April to August. The dominant plant species in the grassland is rough fescue (*Festuca campestris* Rydb.), with Parry’s oatgrass (*Danthonia parryi* Scribn.) and Kentucky bluegrass (*Poa pratensis* L.) as co-dominants.

### Treatments and soil sampling

The grazing trial was established in 1949. Two paddocks with areas of 65 and 32 ha were constructed and stocked with 13 cows and their calves for 6 months (from mid-May to mid-November) to create two stocking rates: 1.2 and 2.4 animal unit months (AUM) ha^−1^. One AUM = 1000 lb (454 kg) of animal consuming 780 lb (354 kg) of air-dried vegetation a month (Understanding AUMs (Animal Unit Months) | UNL Beef). Because the recommended stocking rate was 1.6 AUM ha^−1^, the two stocking rates represent light grazing (75% of the recommended rate) and heavy grazing (150% of the recommended rate), respectively. A 0.8 ha ungrazed paddock was also constructed as a 0 AUM ha^−1^ check treatment. These stocking rates have been maintained every year since 1949. No fertilizer has ever been applied or the vegetation cut during the term of the trial. More details about the experimental site and treatments of this long-term trial can be found in Willms *et al.* [15] and Gao *et al.* [18]. In 2017, 10 soil samples (0–15 cm depth) were collected from each of the 0, 1.2 and 2.4 AUM ha^−1^ paddocks on 30 May (spring grazing season) and 17 October (fall grazing season) and sieved to pass through a 2 mm sieve. The samples were predominantly bulk soil because they were sampled in the spaces between plants, and visible plant roots were removed. The samples for DNA extraction were frozen at −20 °C, those for enzyme analysis were stored at 4 °C, and those for permanganate-oxidizable C (POXC) and microbial biomass C (MBC) analysis were air-dried and stored at 4 °C.

### Soil C fractions: POXC and MBC

Permanganate-oxidizable (active or labile) C was extracted with 0.02 M KMnO_4_, and its absorbance was read with a spectrophotometer at 550 nm wavelength [20]. We measured MBC using the substrate-induced respiration method [21]: we dissolved 300 mg of glucose in 4.5–6.0 mL water and added the solution to 50 g of air-dry soil to bring it to 50% water-holding capacity. The exact amount of water added depended on the pre-determined water content and water-holding capacity of the soil. After stir-mixing, we incubated the soil in a 1 l jar for 3 h at 22 °C and used gas chromatography to measure the amount of CO_2_ that accumulated in the head space.

### DNA extraction, sequencing and bioinformatics

Soil DNA was extracted with the Qiagen DNeasy PowerLyzer PowerSoil kit (Qiagen, Toronto, Ontario) according to the manufacturer’s instructions. Details of amplicon library preparation and MiSeq sequencing procedures used are described by Zhang *et al.* [16]. A summary is given here. To prepare the amplicon library, two PCR reactions were performed. For the first PCR reaction, the V4 hypervariable region of the prokaryotic (bacterial and archaeal) 16S rRNA gene was amplified using the primer set 515F/806R [22]. The second PCR reaction added barcodes to each sample and the Illumina sequencing adapters. After the second amplification, PCR products were quantified, and then multiple samples were pooled together to form a 16S rRNA amplicon library in equal proportion based on their molecular weight. The amplicon library was purified, and the average size and quantity were assessed. Sequencing of the library was done on an Illumina MiSeq platform at the Génome Québec Innovation Centre at McGill University (Montréal, Canada).

Using QIIME2 (v. 2022.11) [23], the 16S samples were demultiplexed and denoised. We then used DADA2 [24] for trimming and clustering the sequences and then outputting the feature table. Taxonomy was assigned with a Naïve Bayes classifier trained for the 16S rRNA gene target region against the silva 138, 99% operational taxonomic unit (OTU), reference database [25]. The resulting tables were further filtered to exclude mitochondrial and chloroplast contamination.

The data were exported and used in the Marker-gene Data Profiling module of the online platform MicrobiomeAnalyst for further bioinformatics analysis (https://www.microbiomeanalyst.ca/) [26,27]. Sequences were filtered for low counts and low variance before analysis. The relative microbial abundances at various classification levels (phylum to genus) were calculated. We calculated the following *α*-diversity indices at the OTU level: ACE (abundance-based coverage estimator), Chao1, Fisher, Shannon and Simpson after rarefying the data to the minimum library size. *β*-Diversity was visualized using Principal Coordinate Analysis (PCoA) on a Bray–Curtis dissimilarity index and group differences in microbial community structures were assessed for statistical significance using permutational multivariate ANOVA (PERMANOVA), all in MicrobiomeAnalyst. Linear discriminant analysis effect size (LEfSe) methods were also used to visualize the differential abundances of the microbial taxa between the experimental treatments.

### Soil enzyme activities

The activities of *β-*glucosidase (C cycling)*, N-*acetyl*-β*-glucosaminidase (NAG) (N cycling) and acid phosphomonoesterase (P cycling) were measured using microplate fluorimetric assays [28] as described by Lupwayi *et al.* [29]. These assays were based on the detection of 4-methylumbelliferone (MUF) released by the enzymatic hydrolysis of MUF-labelled substrates incubated with soil at the optimal pH of each enzyme. For arylsulphatase activity, a bench-scale assay was used to colorimetrically measure the *p*-nitrophenol released by the enzyme after incubating 1 g soil with buffered (pH 6.0) *p*-nitrophenyl-*β*-*d*-glucoside [30].

### Statistical analysis

The soil C fractions, relative abundances of the prokaryotic phyla, the *α*-diversity indices MBC and enzyme activity data were statistically analysed by ANOVA as a stocking rate *x*-grazing season factorial in a Repeated Measures design with ten replicates, with grazing season as the repeated measure. The 5% level of significance was used to determine statistical significance, and the means were separated by the least significant difference test when statistical significance was detected. The relationships between the composition of the microbial genera and enzyme activities were assessed through Pearson correlation analysis, and some of the relationships were modelled with regression analysis when the correlations were significant at the 5% significance level.

## Results

### Soil C fractions

**Fig. 1.**
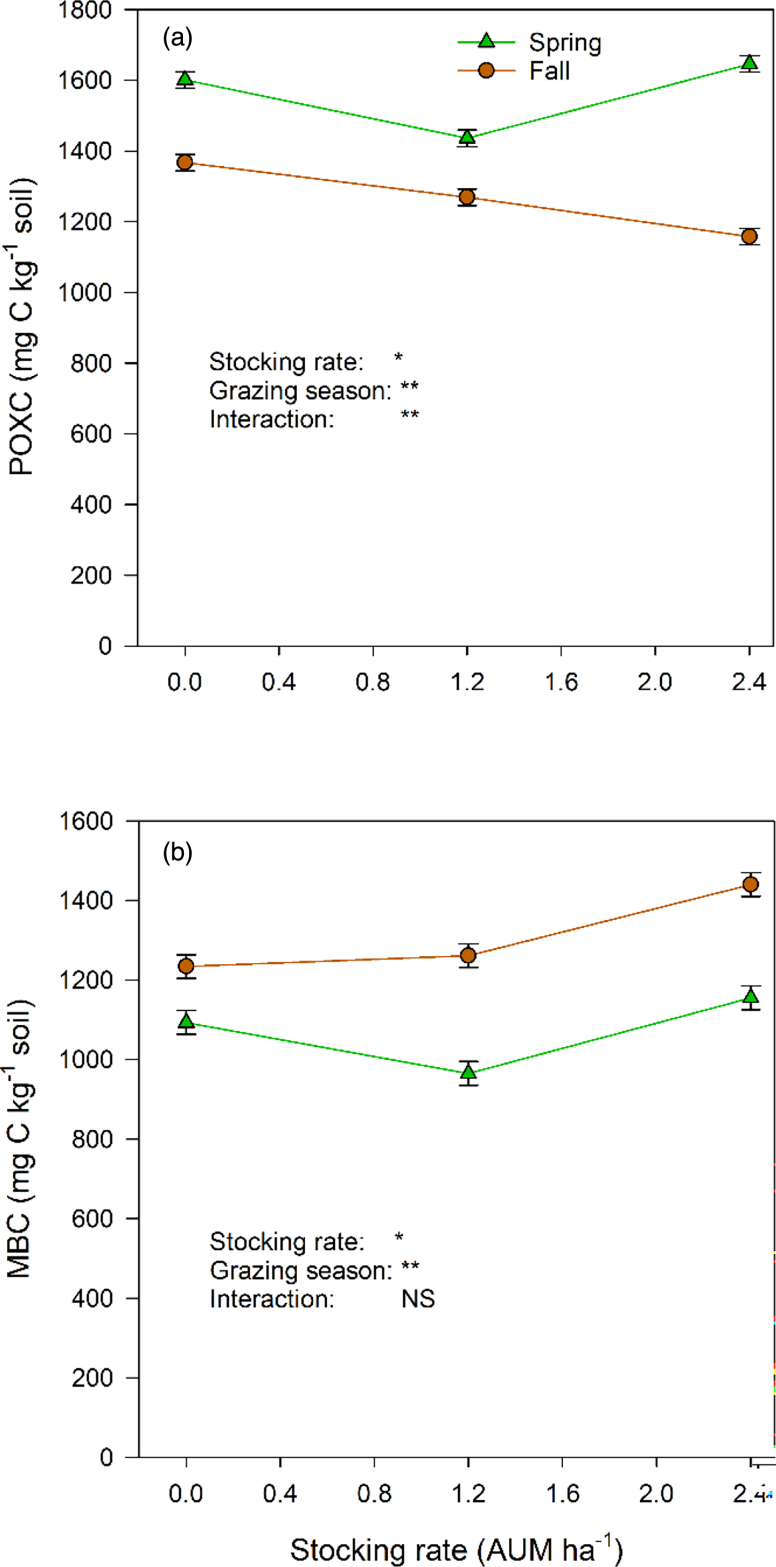
Soil POXC and MBC at different cattle stocking rates in spring and fall.*p<0.05, **p<0.01. NS, not significant.

Relative to the ungrazed treatment (0 AUM ha^−1^), soil POXC was lower with light grazing (1.2 AUM ha^−1^) but higher with heavy grazing (2.4 AUM ha^−1^) in spring, but it declined with increasing cattle stocking rate in fall (Fig. 1a). Spring had greater soil POXC than fall. Although the interaction between stocking rate and sampling time was not significant, soil MBC in spring followed the same pattern as POXC but increased with increasing stocking rate in fall (Fig. 1b). Soil MBC was greater in fall than in spring.

### Prokaryotic community composition

The most abundant prokaryotic phylum (averaged over all treatments) was *Acidobacteriota* (29% relative abundance), followed by *Actinomycetota* (18%) (Fig. S1, available in the online Supplementary Material of this article). At the genus level, the most abundant were *RB41* (11.2%) and *Gaiella* (1.8%) (Fig. S2). The two genera were from the two predominant phyla: *Acidobacteriota* and *Actinomycetota*, respectively.

**Fig. 2.**
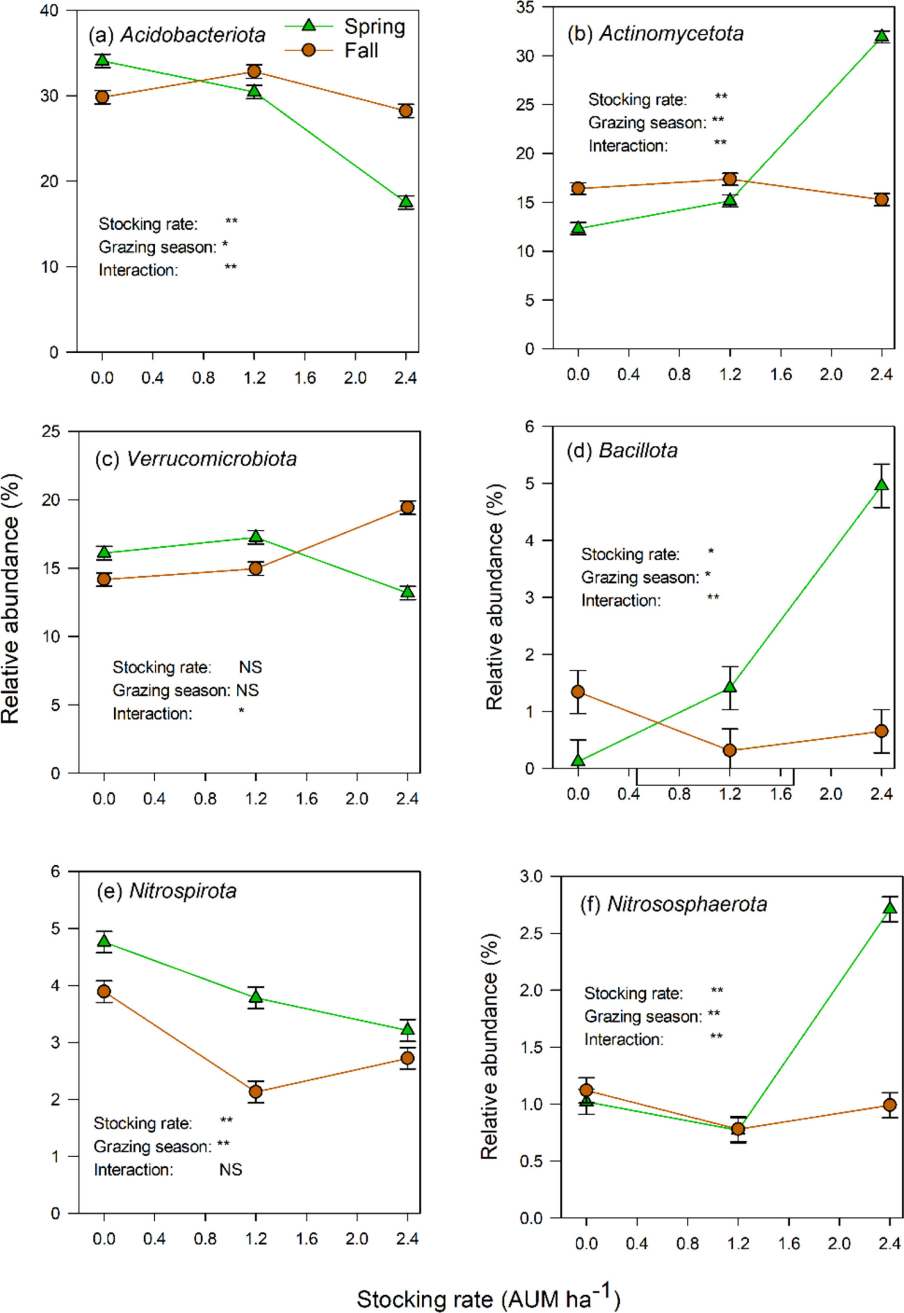
The relative abundances of soil prokaryotic (bacterial and archaeal) phyla at different cattle stocking rates in spring and fall. NS. not significant. *p<0.05, **p<0.01.

In most cases, light grazing had little effect on the relative abundances of soil prokaryotic communities at the phylum level (Fig. 2a–f). Heavy grazing had divergent effects, particularly in spring: it enriched the copiotrophic phyla *Actinomycetota* (Fig. 2b), *Bacillota* (Fig. 2d) and *Nitrososphaerota* (Fig. 2f) at the expense of the oligotrophic phyla *Acidobacteriota* (Fig. 2a), *Verrucomicrobiota* (Fig. 2c) and *Nitrospirota* (Fig. 2e). Consequently, the copiotrophs were relatively more abundant than the oligotrophs in spring, but the reverse was true in fall.

### Prokaryotic community diversity

The indices of *α*-diversity were highest with heavy grazing and lowest with light grazing, irrespective of the grazing season. Thus, the Chao 1, ACE and Fisher indices, which are measures of richness, were in the order: 2.4 AUM ha^−1^ >1.2 AUM ha^−1^ ≥0 AUM ha^−1^ (Table 1). The Shannon index, which is a composite measure of richness and evenness, was not affected by grazing. All the diversity indices were greater in spring than in fall.

**Table 1.** Soil prokaryotic *α*-diversity at different cattle stocking rates in the spring and fall

Treatment	*α*-Diversity index			
	Chao 1	ACE	Fisher	Shannon (H’)
Stocking rate (AUM ha^−1^)				
0	340a, b†	342a,b	121a,b	5.24a
1.2	299b	302b	103b	4.99a
2.4	384a	396a	137a	5.28a
se*	19.5	20.1	8.3	0.093
Grazing season				
Spring	382a	391a	137a	5.31a
Fall	301b	303b	104b	5.03b
se	22.3	23.1	9.2	0.090
Interaction	ns‡	ns	ns	ns

* se = standard error.

†Means followed by the same letter are not significantly different at the 5% significance level.

‡ns = not significant at the 5% significance level.

**Fig. 3.**
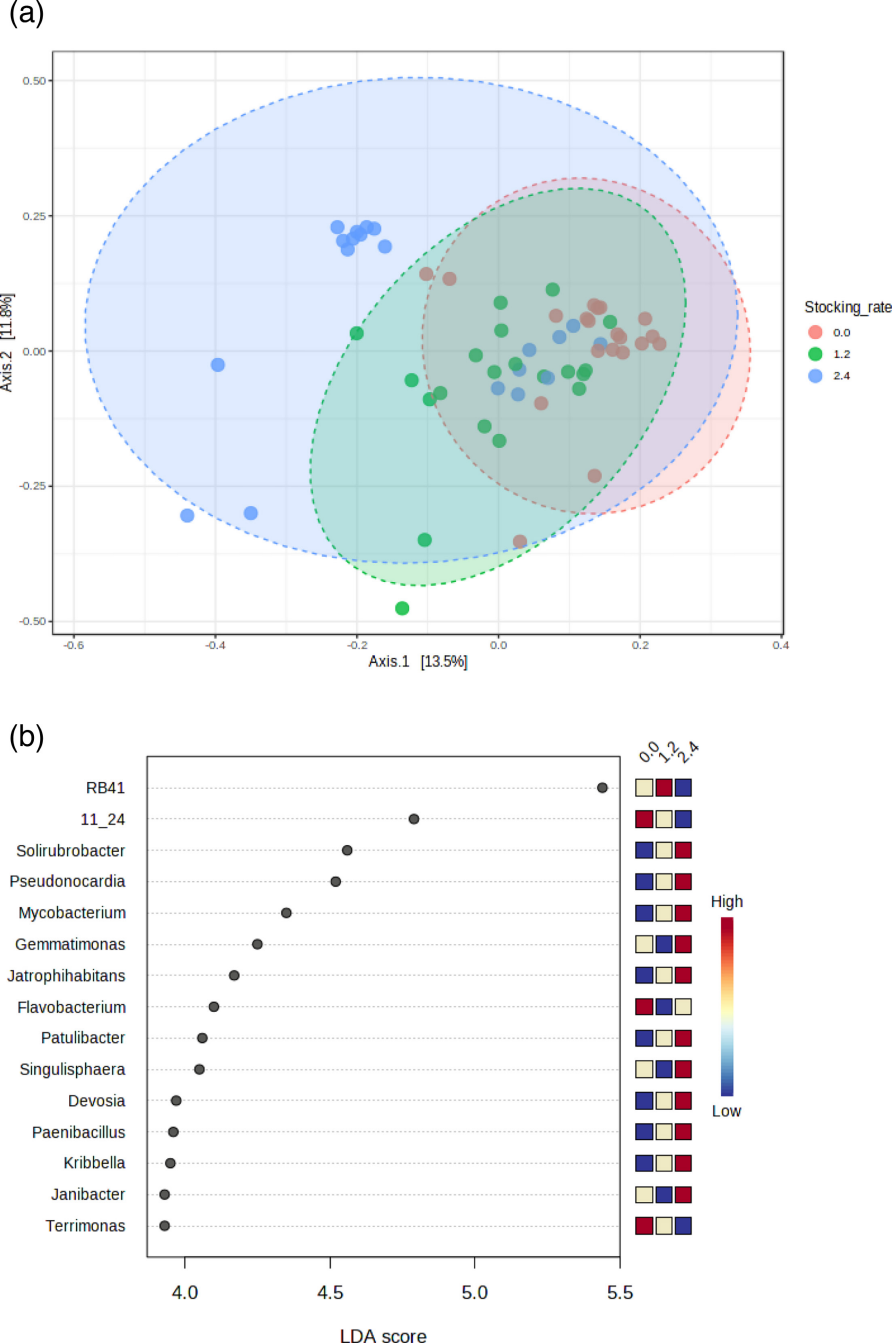
PCoA showing the cattle stocking impacts on the *β*-diversity of the soil prokaryotic communities (**a**) (PERMANOVA *F*-value = 3.4345, *r*^2^ = 0.10755 and *P* = 0.001) and the differential abundances of the prokaryotic genera (determined by LEfSe) at different stocking rates (**b**). LDA, linear discriminant analysis score in LEfSe.

**Fig. 4.**
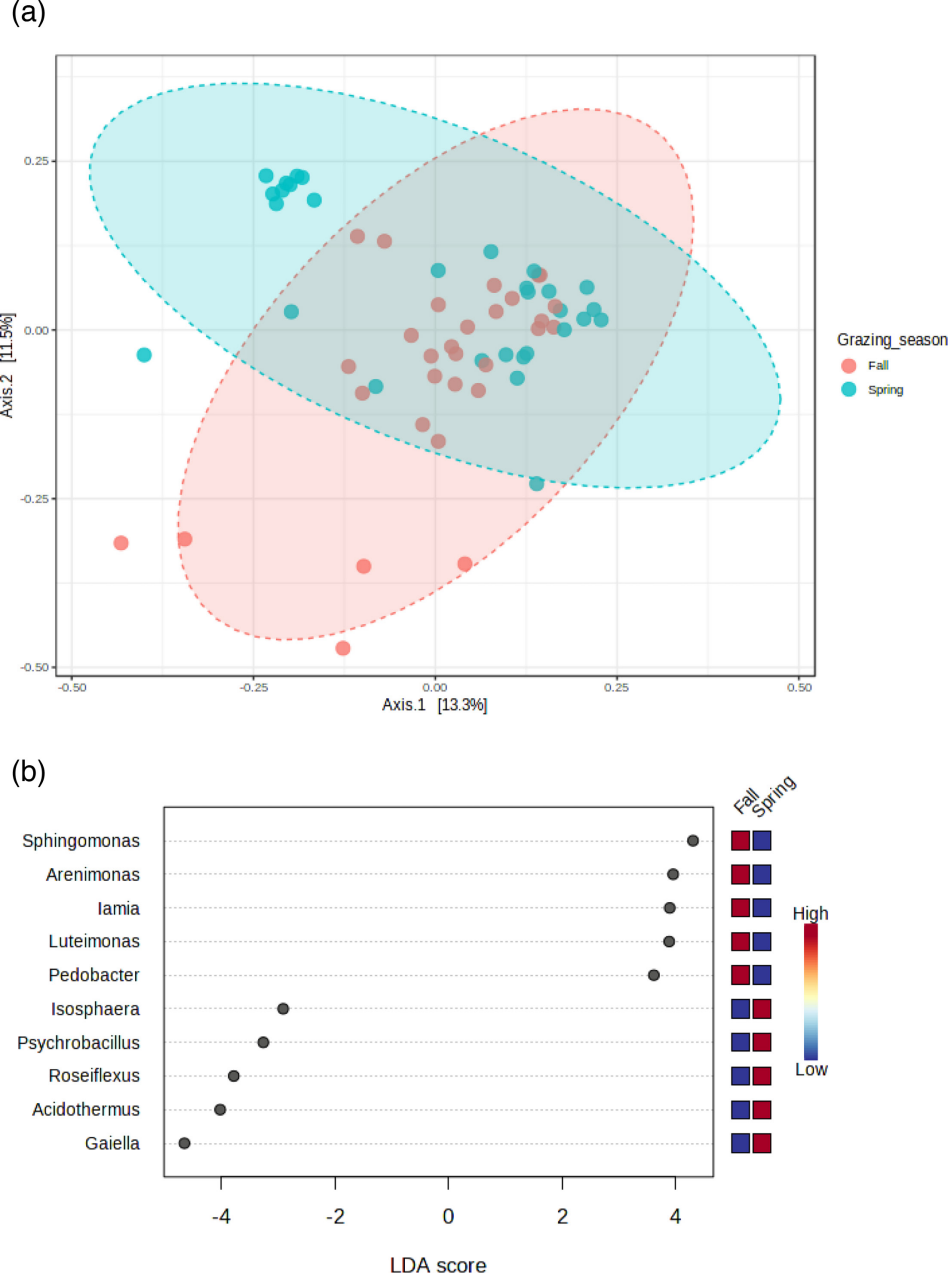
PCoA showing the grazing season impacts on the *β*-diversity of the soil prokaryotic communities (**a**) (PERMANOVA *F*-value = 2.5588, *r*^2^=0.042252 and *P*=0.001) and the differential abundances of the prokaryotic genera (determined by LEfSe) between the two seasons (**b**). LDA, linear discriminant analysis score in LEfSe.

PCoA analysis of *β*-diversity indicated that the soil prokaryotic community structures at the 2.4 AUM ha^−1^ cattle stocking rate were different from those at 0 and 1.2 AUM ha^−1^ stocking rates, even though there was some overlap with the lower stocking rates (Fig. 3a). LEfSe determined that most of the prokaryotic genera that were enriched at 2.4 AUM ha^−1^ belonged to the copiotrophic phylum *Actinomycetota*, i.e. *Solirobrobacter*, *Pseudonocardia*, *Mycobacterium*, *Jatrophihabitans*, *Patilubacter*, *Kribella* and *Janibacter* (Fig. 3b). Seasonal differences in *β*-diversity were also detected, where the prokaryotic community structures in spring were different from those in the fall (Fig. 4a). Surprisingly, according to LEfSe, all six genera that were more abundant in the fall than spring belonged to copiotrophic phyla: the phylum *Pseudomonadota* for the genera *Sphingomonas*, *Arenomonas* and *Luteimonas*; the phylum *Actinomycetota* for the genera *Nocardioides* and *Iamia*; and the phylum *Bacteroidota* for the genus *Pedobacter* (Fig. 4b). The five genera that were more abundant in spring than fall were a mixture of copiotrophs (the genera *Gaiella* and *Acidothermus* from the phylum *Actinomycetota* and the genus *Psychrobacillus* from the phylum *Bacillota*) and oligotrophs (the genus *Isosphaera* from the phylum *Planctomycetota* and the genus *Roseiflexus* from the phylum *Chloroflexota*).

### Core genera

**Fig. 5.**
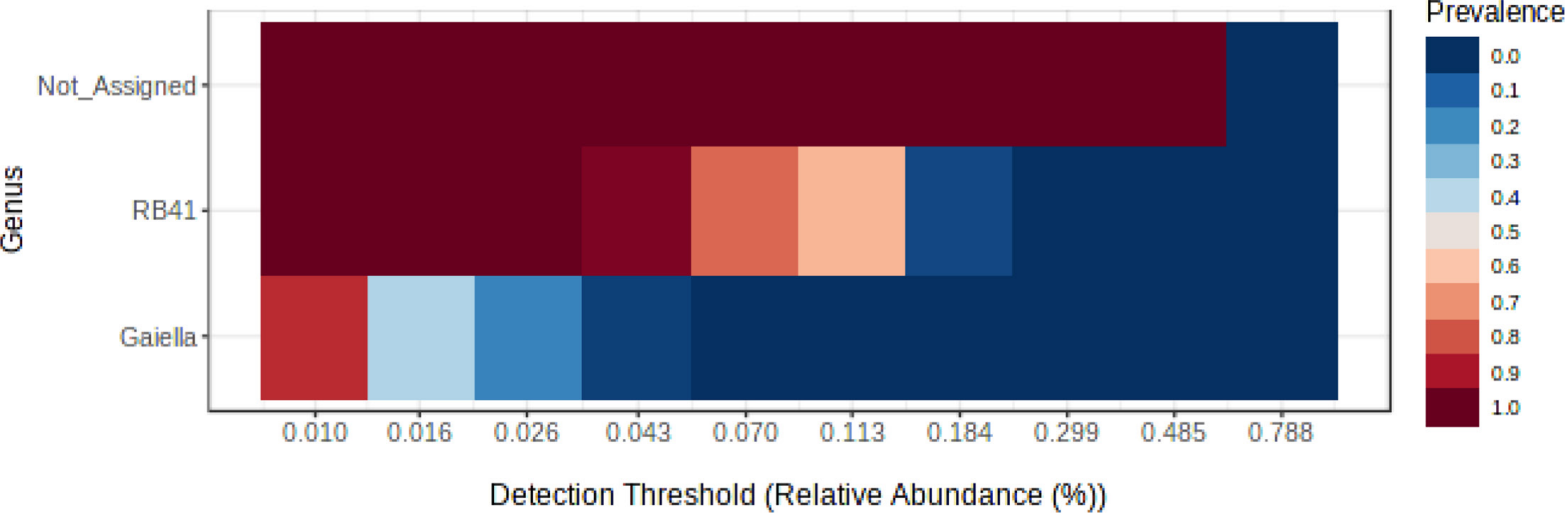
The core prokaryotic genera that were observed in 85% of the soil samples at all cattle stocking rates and both grazing seasons.

Notwithstanding the differential abundances of the different prokaryotic genera between treatments, there were core genera that were present in 85% of the samples: *RB41, Gaiella* and an unassigned genus (Fig. 5). The genera *RB41* and *Gaiella* were also the most abundant in these grassland soils (Fig. S2).

### Soil enzyme activities

**Fig. 6.**
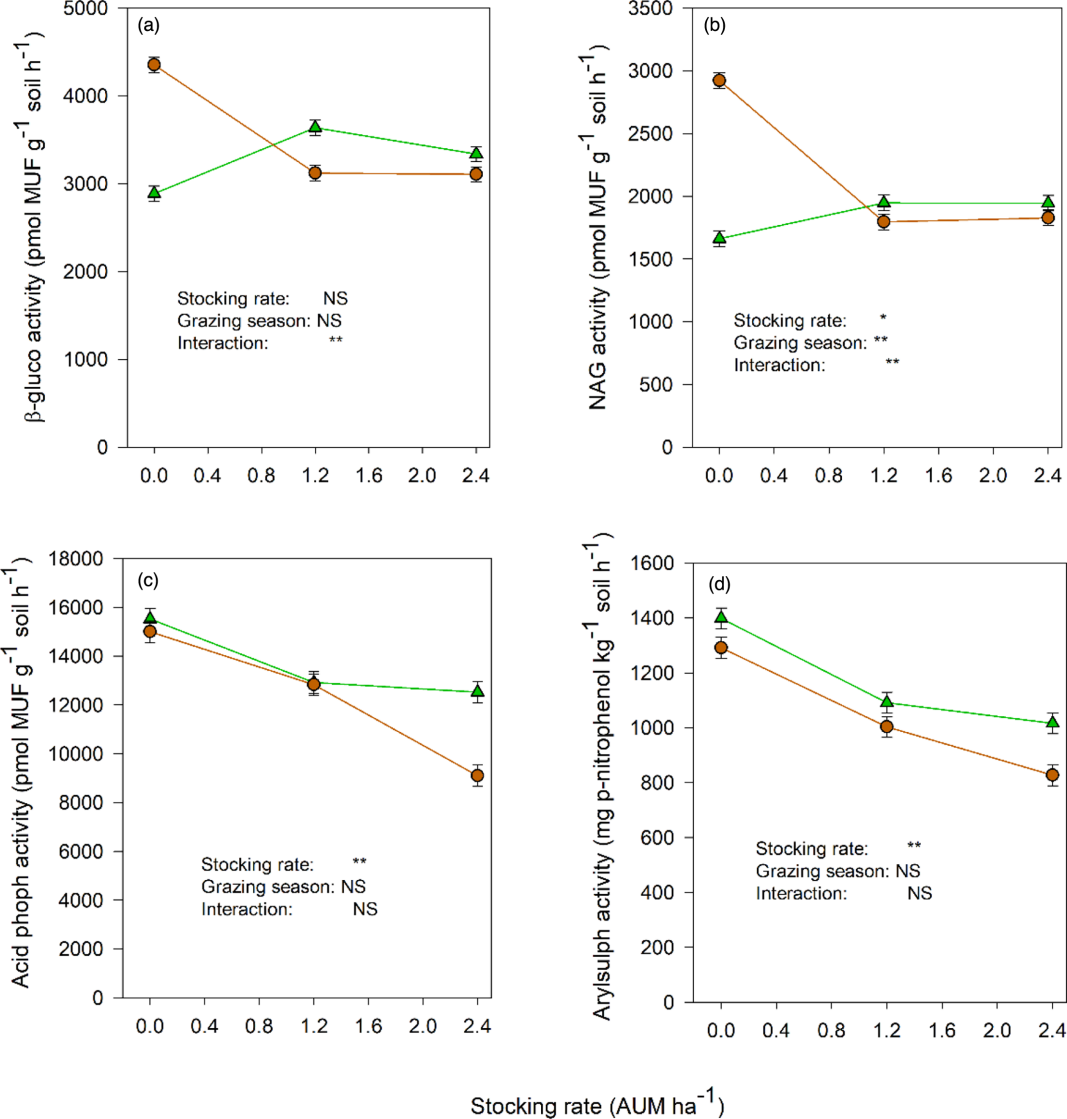
Soil enzyme activities at different cattle stocking rates in spring and fall. NS, not significant. *p<0.05, **p<0.01.

Light grazing increased *β*-glucosidase activity in spring but decreased it in fall, relative to the ungrazed control (Fig. 6a). Heavy grazing did not change this enzyme activity further (i.e. relative to light grazing). A similar pattern was observed with NAG activity, but the increase in spring was not as much as that for *β*-glucosidase activity (Fig. 6b). The activities of acid phosphomonoesterase (Fig. 6c) and arylsulphatase (Fig. 6d) decreased with increasing cattle stocking rate, with no interactions between stocking rate and grazing season. The grazing season did not affect the activities of these two enzymes. The alkaline phosphomonoesterase activity was not affected by either treatment (data not shown).

### Correlations between soil prokaryotic genera composition and enzyme activities

**Fig. 7.**
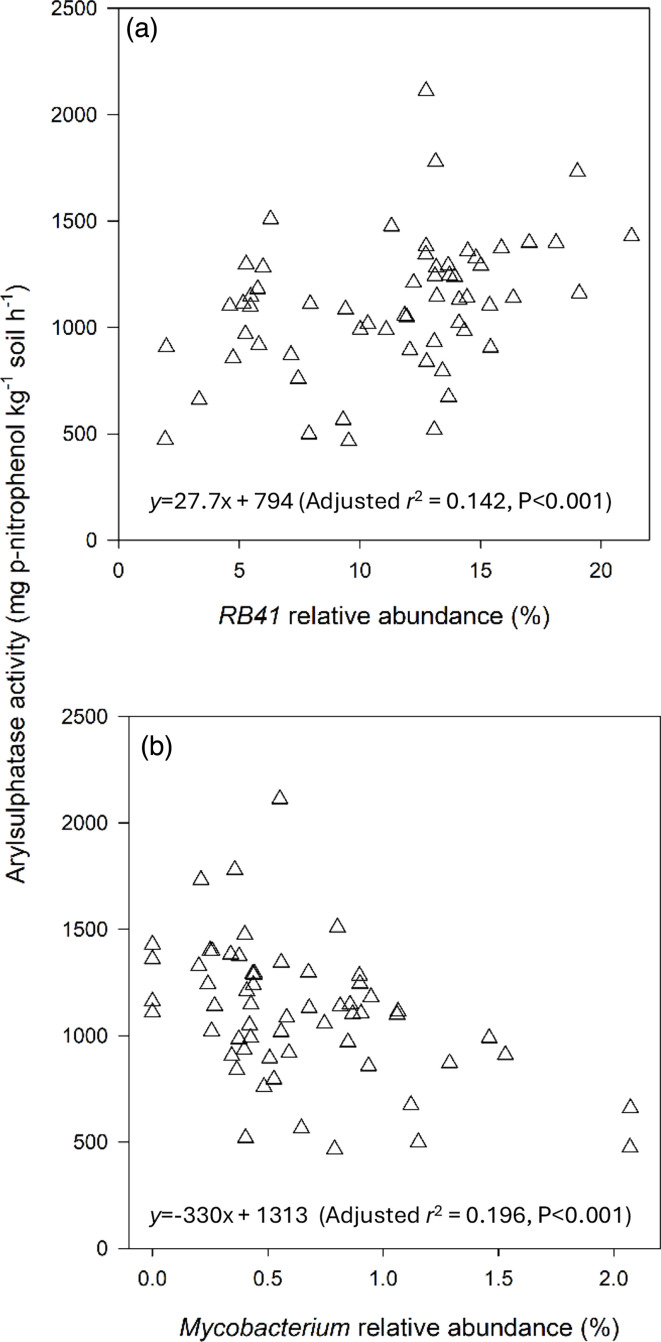
Relationships between arylsulphatase enzyme activities and the relative abundances of the genera *RB41* (**a**) and *Mycobacterium* (**b**).

With the exception of the core genus *RB41* from the oligotrophic phylum *Acidobacteriota,* all significant correlations with the activities of enzymes that mediate C, N, P or S cycling involved genera from the copiotrophic phyla *Actinobacteria* and *Pseudomonadota* (Table 2). The relative abundance of the genus *RB41* was positively correlated with arylsulphatase activity (Table 2 and Fig. 7a). All three significant correlations involving genera from the phylum *Actinomycetota* (the genera *Jatrophihabitans, Mycobacterium* and *Nocadiodes*) were negative (Table 2 and Fig. 7b for *Mycobacterium*). Only correlations with genera from the phylum *Pseudomonadota* were either positive or negative.

**Table 2.** Correlations (*n* = 60) between the relative abundances of prokaryotic genera and enzyme activities. Only genera with at least 0.2% average relative abundance, and with at least one significant correlation, are included. phosph, phosphomonoesterase

Genus(phylum)	*β*-Glucosidase	NAG	Acid phosph	Alkaline phosph	Arylsulphatase
	Correlation coefficient(probability)				
*11_44* (*Pseudomonadota*)	-	-	0.393(0.002)	-	0.392(0.002)
*Bradyrhizobium* (*Pseudomonadota*)	-	-	-	0.263(0.042)	−0.264(0.042)
*H16* (*Pseudomonadota*)	-	-	0.302(0.019)	-	-
*Jatrophihabitans* (*Actinomycetota*)	−0.300(0.020)	-	−0.392(0.002)	-	−0.410(0.001)
*Mycobacterium* (*Actinomycetota*)	−0.283(0.029)	-	−0.399(0.002)	-	−0.458(<0.001)
*Nocardioides* (*Actinomycetota*)	-	-	-	-	−0.273(0.035)
*RB41* (*Acidobacteriota*)	-	-	-	-	0.395(0.002)
*Rhodoplanes* (*Pseudomonadota*)	-	−0.263 (0.043)	-	-	-
*Stenotrophobacter* (*Pseudomonadota*)	-	-	-	0.259(0.046)	-
*Streptomyces* (*Actinomycetota*)	-	-	−0.327(0.011)	-	-

## Discussion

One part of our hypothesis, that grazing intensity would affect the studied soil microbial properties, has been supported by the results. The responses of soil MBC and the composition and diversity of prokaryotic communities to cattle stocking rate were mostly observed with heavy grazing (2.4 AUM ha^−1^) and not light grazing (1.2 AUM ha^−1^). Several studies have also shown negligible detrimental effects of light or moderate grazing on the soil microbiome [10], and other studies have shown stimulatory effects [12,13, 31,33]) and reported that light grazing (0.34 AUM ha^−1^) had the highest soil MBC and attributed the increase in part to improvement of soil aeration by light trampling of the litter layer. Light or moderate grazing has been recommended as an effective practice to maintain ecological services in grasslands [19,34]. When grazing intensities are labelled as light, moderate or heavy, the actual stocking rates should be noted. On the same experimental site as this study, Zhang *et al*. [19] labelled 2.4 AUM ha^−1^ as moderate grazing, but we have labelled it as heavy grazing because it was 150% of the recommended stocking rate of 1.6 AUM ha^−1^. Heavy grazing has been reported to have negative effects on the grassland soil microbiome in many studies, both at this experimental site [19] and elsewhere [7,9]. These negative consequences have been attributed to many factors, including the defoliation of vegetation by grazing, thereby reducing organic C inputs to the soil [9,35] and reducing mulching [36].

The second part of our hypothesis, that the grazing effects would be similar in the two grazing seasons, has not been supported by the results. Heavy grazing (2.4 AUM ha^−1^) resulted in divergent responses of the soil prokaryotes in the spring grazing season, when the relative abundances of copiotrophic phyla increased, while those of oligotrophic phyla decreased. Usman *et al.* [37] reported similar results in a typical steppe of Northern China’s Loess Plateau, especially in summer grazing relative to winter grazing. In our study, this difference was probably due to the soil organic C contents because POXC (active) C increased with heavy grazing (Fig. 1a). Most soil microbes use organic C as a substrate for their metabolism and source of energy. Copiotrophs use labile organic compounds and thrive in environments with high organic C and nutrient contents at the expense of oligotrophs, and vice versa in low organic C and nutrient environments which oligotrophs tolerate better because they can use recalcitrant compounds [38]. This difference in responses was not observed in the fall, where grazing reduced soil POXC, MBC and the relative abundances of most phyla. Soil labile C contents in spring increase due to the addition of rhizodeposits (by growing roots) and plant litter (through cattle trampling) of freshly regenerating (low C/N ratio) vegetation. In the fall, there is little root growth and most of the vegetation is mature (high C/N ratio) and brown.

The β-diversity analysis results showed that the prokaryotic community structure under heavy grazing was different from those in the control and light grazing treatments, and *α*-diversity indices (except the Shannon index) were highest under heavy grazing in both grazing seasons. Zhang *et al.* [19] did not find any responses of these indices to 2.4 AUM ha^−1^ cattle stocking rate on the same experimental site, but they reported a decrease in the Simpson index at 4.8 AUM ha^−1^. In agreement with our results, many other studies have reported increases in soil microbial diversity with grazing [37,39,42] but the reverse has also been reported [32]. Therefore, the effect of grazing on soil microbial *α*-diversity is likely context-dependent. Wu *et al.* [42] and Eldridge *et al.* [9] speculated that one reason for the grazing-induced increase in soil microbial diversity could be the result of reduced competition between taxa due to the detrimental effect of grazing on soil microorganisms, as observed for MBC in our study. Another possible reason is that grazing increases plant diversity (by reducing dominant plant species in ungrazed pastures), which translates into increased below-ground diversity of roots, root exudates and plant litter that increases soil microbial diversity [43]. Another factor could be cow dung. The spring soil samples were taken almost immediately after animals were placed in the field each year, while the autumn samples were taken after an entire season of stocking was complete. More dung was presumably added to the soil with increasing stocking rates, and it may have affected soil microbial diversity and the soil microbial parameters measured.

The soil enzyme activities in this native grassland were 4–10× the activities reported in arable systems of the Canadian prairies [44]. It is a reflection of the high organic C contents in grassland soils. In a 2016 study on this experimental site, the soil organic C content in the ungrazed control treatment was 102 g kg^−1^ soil [16], which is at least 4× the amounts in the arable soils of the Canadian prairies [44]. Even the soil POXC contents reported here (Fig. 1) and the 2016 study [19] are very high relative to those observed in arable soils [44]. The activities of the enzymes that catalyse the recycling of C (*β*-glucosidase) and N (NAG) differed from the activities of P- and S-mobilizing enzymes in their responses to grazing: the latter declined with increasing grazing intensity in both the spring and fall, but the former declined only in the fall. The negative effects of grazing on enzyme activities have been reported in other studies in Alberta [45] and elsewhere [41,46]. It was mostly the relative abundances of copiotrophic bacteria that were related (negatively or positively) to the activities of these enzymes (Table 2), suggesting the importance of these bacteria in C and nutrient cycling in the spring when the relative abundances of copiotrophic prokaryotes increased (Fig. 2b, d, f).

The bacterial genera *RB41* (from the phylum *Acidobacteriota*) and *Gaiella* (from the phylum *Actinomycetota*) were present in almost all (85%) of the soil samples of this study (Fig. 5), suggesting that they were suited to the grassland ecosystem and perhaps providing key services. The genus *RB41* has been reported to be one of the dominant genera in other grasslands [47,49], as has the genus *Gaiella* [32,50]. One service that was provided by the genus *RB41* in our study was probably S-acquisition because its relative abundance was positively correlated with the activity of the S-mobilizing enzyme arylsulphatase. The relative abundance of the genus *Gaiella* was not correlated with any enzyme activity.

## Conclusion

Moderate grazing (75% of the recommended stocking rate) had mostly no effects on the composition of the soil microbiome, but the effect of heavy grazing (150% of the recommended rate) depended on the grazing season. In spring, heavy grazing increased the relative abundances of copiotrophic prokaryotes *Actinomycetota, Bacillota* and *Nitrososphaerota* while reducing those of oligotrophic prokaryotes *Acidobacteriota, Verrucomicrobiota* and *Nitrospirota*. The activities of P- and S-mobilizing soil enzymes decreased with increasing cattle stocking rate in both seasons, but the activities of the enzymes that mediate C and N cycling decreased only in the fall. One of the two core bacterial genera in this soil, *RB41* (phylum *Acidobacteriota*), was positively correlated with the activity of the S-mobilizing enzyme. These results suggest that since these grasslands are mostly used for grazing, light grazing should be the recommended management practice for minimal negative effects on the soil microbiome. The results also highlight the need to consider the grazing season when evaluating grazing effects on the soil microbiome.

## supplementary material

10.1099/mic.0.001517 Uncited Fig. S1.

## Abbreviations

ACE: abundance-based coverage estimator
AUM: animal unit months
LEfSe: linear discriminant analysis effect size
MBC: microbial biomass C
MDP: Marker-gene Data Profiling
MUF: 4-methylumbelliferone
NAG: N-acetyl-β-glucosaminidase
PCoA: Principal Coordinate Analysis
PERMANOVA: permutational multivariate ANOVA
POXC: permanganate-oxidizable C
