# Supplementary material for: Divergent responses of the native grassland soil microbiome to heavy grazing between spring and fall
Source: Microbiology (Reading). 2024 Nov 26;170(11):001517. doi: 10.1099/mic.0.001517 (PMC11893364; doi:10.1099/mic.0.001517)

Figure S1. The relative abundances of soil prokaryotic phyla at different cattle stocking rates, in spring and fall. AUM = Animal unit months.

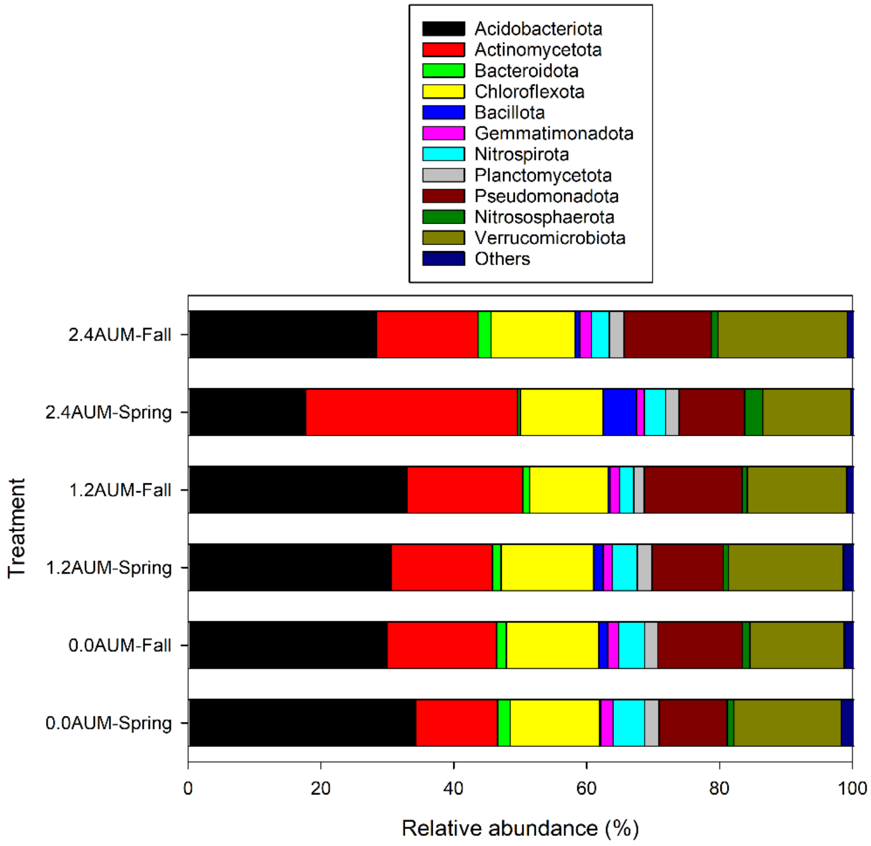

Figure S2. The relative abundances of soil prokaryotic genera at different cattle stocking rates, in spring and fall. Only the genera with at least 0.2% average relative abundance are included. The majority of the un-graphed taxa were un-assigned to a genus. AUM = Animal unit months.

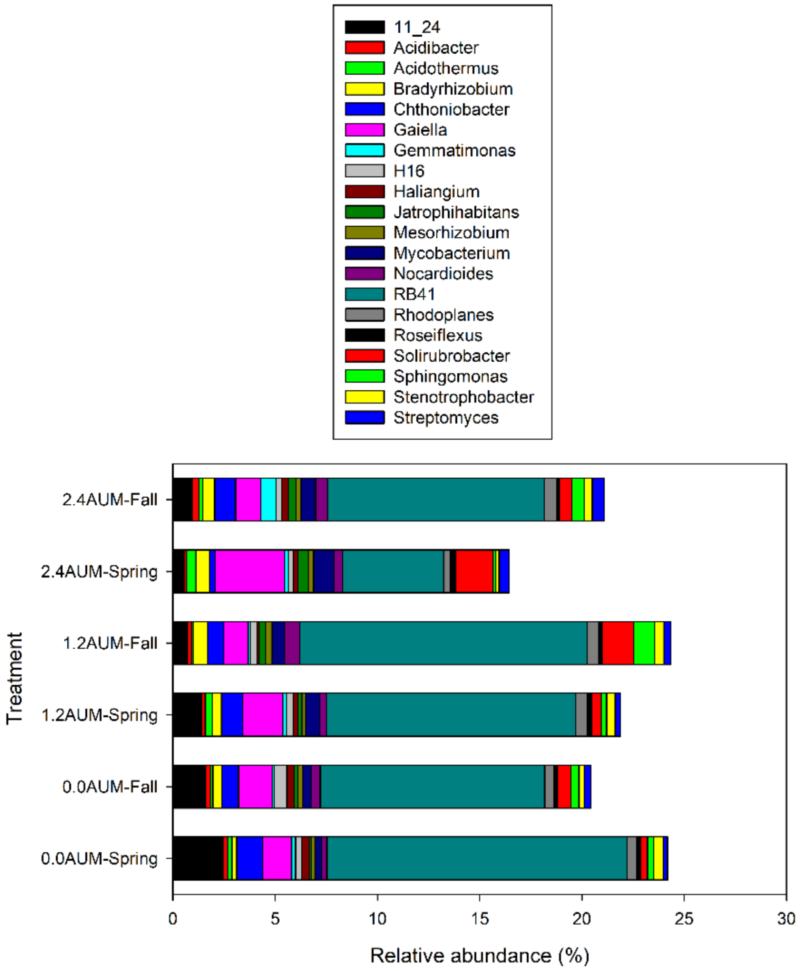

Supplement: Uncited Fig. S1. [file mic-170-01517-s001.pdf]
